# Mapping knowledge landscapes and emerging trends of sonodynamic therapy: A bibliometric and visualized study

**DOI:** 10.3389/fphar.2022.1048211

**Published:** 2023-01-09

**Authors:** Zhenjiang Wu, Kunming Cheng, Zefeng Shen, Yanqiu Lu, Hongtao Wang, Guolei Wang, Yulin Wang, Weiguang Yang, Zaijie Sun, Qiang Guo, Haiyang Wu

**Affiliations:** ^1^ Department of Thoracic Surgery, Henan Provincial Chest Hospital, Zhengzhou University, Zhengzhou, China; ^2^ Department of Intensive Care Unit, The Second Affiliated Hospital of Zhengzhou University, Zhengzhou, Henan, China; ^3^ Department of Graduate School, Sun Yat-Sen Memorial Hospital, Sun Yat-sen University, Guangzhou, China; ^4^ Department of Graduate School of Tianjin Medical University, Tianjin, China; ^5^ Department of Clinical College of Neurology, Neurosurgery and Neurorehabilitation, Tianjin Medical University, Tianjin, China; ^6^ Department of Orthopaedic Surgery, Xiangyang Central Hospital, Affiliated Hospital of Hubei University of Arts and Science, Xiangyang, China; ^7^ Department of Orthopaedics, Baodi Clinical College of Tianjin Medical University, Tianjin, China; ^8^ Duke University School of Medicine, Duke Molecular Physiology Institute, Duke University, Durham, NC, United States

**Keywords:** sonodynamic therapy, ultrasound, cancer, nanoparticle, bibliometric, data visualization

## Abstract

**Background:** Ultrasound-triggered sonodynamic therapy (SDT), as a non-invasive approach, has attracted considerable attention in a wide variety of malignant tumors and other diseases. Over the past 2 decades, the number of scientific publications on SDT has increased rapidly. However, there is still a lack of one comprehensive report that summarizes the global research trends and knowledge landscapes in the field of SDT in detail. Thus, we performed a bibliometric analysis on SDT from 2000 to 2021 to track the current hotspots and highlight future directions.

**Methods:** We collected publications on SDT research from the Web of Science Core Collection database. The annual number of publications and citations, major contributors, popular journals, international collaborations, co-cited references and co-occurrence keywords were analyzed and visualized with CiteSpace, VOSviewer, and R-bibliometrix.

**Results:** A total of 701 publications were included. The annual publication output increased from 5 in 2000 to 175 in 2021, and the average growth rate was 18.4%. China was the most productive country with 463 documents (66.05%), and Harbin Medical University was the most prolific institution (N = 73). *Ultrasound in Medicine and Biology* published the most papers related to SDT. *Materials Science*, and *Chemistry* were the research areas receiving the most interest. All the keywords were divided into four different clusters including studies on mechanisms, studies on drug delivery and nanoparticles, studies on cancer therapy, as well as studies on ultrasound and sonosensitizers. In addition to nanomaterials-related studies including nanoparticles, mesoporous silica nanoparticles, nanosheets, liposomes, microbubble and TiO_2_ nanoparticle, the following research directions such as immunogenic cell death, metal-organic framework, photothermal therapy, hypoxia, tumor microenvironment, chemodynamic therapy, combination therapy, tumor resistance, intensity focused ultrasound, drug delivery, and *Staphylococcus aureus* also deserve further attention and may continue to explode in the future.

**Conclusion:** SDT has a bright future in the field of cancer treatment, and nanomaterials have increasingly influenced the SDT field with the development of nano-technology. Overall, this comprehensive bibliometric study was the first attempt to analyze the field of SDT, which could provide valuable references for later researchers to better understand the global research trends, hotspots and frontiers in this domain.

## Introduction

Cancer is considered to be one of the predominant causes of human mortality worldwide, with an increasing number of cases over recent years. According to forecasts, annual cancer-related deaths are projected to increase to 11.5 million by 2030 globally (Mathers and Loncar, 2006). At present, it is generally assumed that surgery, chemotherapy and radiotherapy are the most fundamental and effective antitumor strategies (Mellman et al., 2011; Hartmann et al., 2017). However, most of these approaches possess some limitations. Surgical resection could temporarily remove the primary lesion, but it is difficult to completely remove due to the high migratory capacity of tumor cells. The main issue with both chemotherapy and radiotherapy is the absence of selectivity, which in turn, won’t only damage cancer cells but also cause unwanted toxicity to normal tissues (Ahmad et al., 2019). Thus, it is of great significance to find novel anti-tumor approaches and explore effective therapeutic schemes.

Sonodynamic therapy (SDT) is a relatively new method for non-invasive cancer treatment using similar principles to photodynamic therapy (PDT), which involves the synergistic effect on tumor killing by the association of ultrasound and sonosensitizer drugs (Rosenthal et al., 2004; Son et al., 2020). Nevertheless, unlike PDT, it could focus ultrasound energy to target on the deeply located tumor site, which overcomes the shortcoming of the shallow penetration depth of light sources in PDT (Trendowski, 2014; Rengeng et al., 2017). Despite numerous investigations on SDT, the precise underlying mechanism wasn’t fully elucidated (Rosenthal et al., 2004; Bai et al., 2012; Zhu et al., 2022). Generally speaking, SDT requires a sensitizing agent, ultrasound energy and oxygen molecules to generate ROS, such as singlet oxygen (^1^O_2_), hydroxyl radicals (•OH), and superoxide radicals (·O2^−^), which mediate cellular toxicity. To date, several studies have demonstrated that many chemical products such as porphyrin and its derivatives, cisplatin, fluoroquinolone agents, etc., were able to act as sonosensitizers (Chen et al., 2014; Pang et al., 2016). Meanwhile, with the rapid advancement of nanotechnology in recent years, certain rationally designed biocompatible nanomaterials have shown great potential in SDT, which further motivated the development of SDT against cancer (Qian et al., 2016; Mohapatra et al., 2021; Guo et al., 2022; Zhu et al., 2022).

Many previous studies have validated the efficacy of SDT on multiple types of cancers including prostate cancer (Hadi et al., 2021), glioma (Wang et al., 2017; Bunevicius et al., 2022), breast cancer (Chen et al., 2021; Huang et al., 2022), head and neck neoplasms (Hajmohammadi et al., 2021), and so on. In addition to its use in treating solid tumors, SDT also has high application value in the fields of atherosclerosis (Geng et al., 2018), leukemia (Trendowski, 2015), proliferative scars (Li et al., 2015), rheumatoid arthritis (Li et al., 2021), as well as fighting against pathogenic microorganism (Pang et al., 2020). Nevertheless, despite the promising results of this technique, there are still many questions such as the setting and management of ultrasound waves’ physical parameters, the improvement of sonosensitizers that remain to be addressed (Wood and Sehgal, 2015; Lafond et al., 2019). Data on technical performance and clinical outcomes are also lacking. Motivated by these limitations, growing scientific effort is put into the investigation of its exact mechanisms and clinical trials are gradually unfolding. In the past few years, a considerable number of studies focusing on the theme of SDT have been published. However, there is still a lack of one comprehensive report that summarizes the global research trends and knowledge landscapes of this field in detail. Although several reviews and meta-analysis have been published to summarize the research progress on SDT, insightfulness of such summaries is relatively limited as the experience of many researchers is usually limited to a particular direction and lacks a holistic understanding of the entire field. In addition, reviews and meta-analysis are also unable to provide several key information such as the main contributors, and the developmental process of research hotspots. Of note, bibliometrics, as a novel analytical approach, could make up these deficiencies.

Bibliometrics is a branch of library and information science that integrates mathematical and statistical methods to quantitatively analyze the published scientific literature, and then discusses its structure, characteristics and change trends in a certain period (Cheng et al., 2022a; Li et al., 2022a; Shen et al., 2022a; Cheng et al., 2022b). In recent years, with more and more free open-source software such as CitNetExplorer, CiteSpace, HistCite, VOSviewer, and Bibliometrix have been developed, bibliometrics relying on literature databases has been frequently applied in biomedical field (Synnestvedt et al., 2005; van Eck and Waltman, 2010; Aria and Cuccurullo, 2017; Liu et al., 2022). Take ultrasound technology as an example, several bibliometric studies have analyzed the publication trend and research hotspots in the field of spinal ultrasound (Zhai et al., 2017), endoscopic ultrasound (Chen et al., 2022), ultrasound microbubble (Wu et al., 2021a), ultrasound induced blood-brain barrier opening (Wu et al., 2021b). In addition, in the field of PDT, Cheng and colleagues have collected 5804 documents regarding cancer PDT published from 2000 to 2021 by using bibliometric approach. Then they pointed out that nanotech-based PDT and enhanced PDT were research focuses in this area (Cheng et al., 2022c). Whereas, to our knowledge, no bibliometric study has yet examined the global research trends in the field of SDT.

To fill this gap, we conducted a comprehensive bibliometric analysis based on publications related to SDT since 2000. In this study, we mainly address the following questions: i) global publication and citation trends; ii) major contributors including countries/regions, institutions, and funding agencies; iii) most popular journals; iv) main research directions and focuses in current work; v) potential research hotspots and frontiers in the future. Collectively, we hope this study could provide new ideas and perspectives for researcher’s future work in the SDT area.

## Methods and materials

### Data source

Web of Science Core Collection (WoSCC) is one of the most well-known and authoritative citation databases, covering more than 21,100 peer-reviewed, high-quality scholarly journals and over 250 disciplines (Mbaidjol et al., 2019). Compared with the other databases, WoSCC is generally considered to be the most suitable online database for bibliometric analysis (Yeung, 2019; Yeung et al., 2021). Therefore, in this research, data were retrieved and downloaded from the Science Citation Index-Expanded of WoSCC.

### Search strategy

**FIGURE 1:**
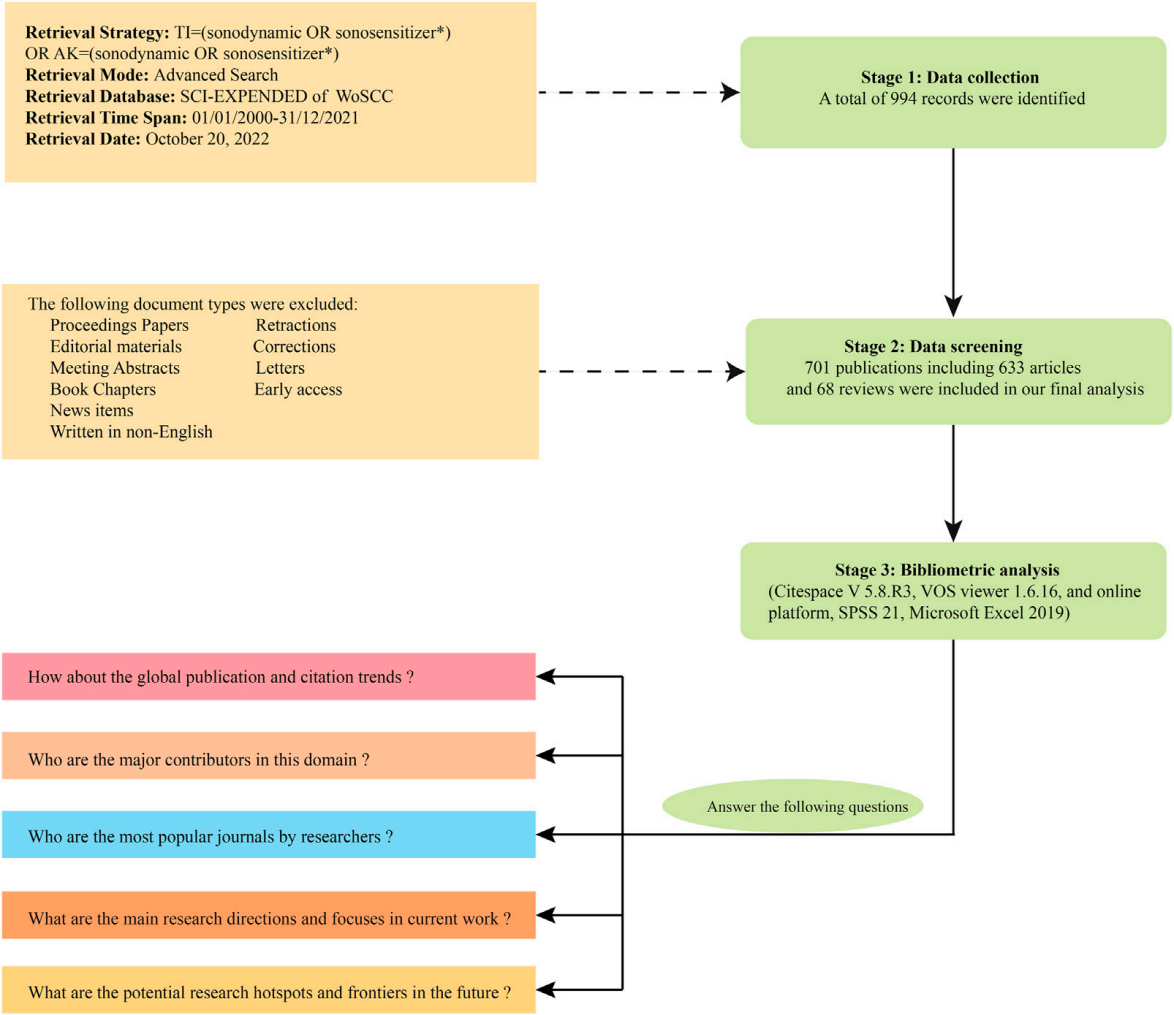
Flowchart of literature screening and data analysis.

All the search works were completed within 1 day (20 October 2022) to avoid the possible bias caused by daily updating from WoSCC database. All the terms were searched in titles (TI) and author keywords (AK). The search formula was set as follows: TI = (sonodynamic or sonosensitizer*) or AK = (sonodynamic or sonosensitizer*). The wildcard character (*) is a character that can be substituted for one or more other characters. For example, “sonosensitizer*” would return the terms of “sonosensitizer” or “sonosensitizers”. The retrieval time span was limited to 01/01/2000–31/12/2021. Only original articles or reviews published in English were enrolled. All the records were double checked by two independent investigators to remove duplicates. Then data involving full records and cited references were downloaded in txt format and tab-delimited format, respectively. The detailed literature search strategies and screening process were shown in Figure 1.

### Statistical analysis

Microsoft office Excel 2019 (Microsoft, Redmond, WA, United states) and SPSS (IBM SPSS Statistics 21, Inc. Chicago, IL, United states) were used to analyze the data. The online website (https://www.highcharts.com.cn) was used to generate graphs, such as in Figures 3C, 4B. Firstly, all the exported data including the number of publications and citation frequencies, countries/regions, institutions, funding agencies, journals, research areas were summarized into an excel spreadsheet. Curve fitting was performed using Excel 2019, and the best fit curve was generated based on the coefficient of determination (*R* ^2^). According to the formula described by Cheng et al. (Cheng et al., 2022c) we calculated the average growth rate of publications from 2000 to 2021. In addition, Pearson’s correlation coefficient was calculated for the correlation of annual publications and citations by using SPSS 21. As for the journal information, impact factor (IF) and quartile in category (Q1/Q2/Q3/Q4) were acquired from 2021 Journal Citation Reports (JCR). The H-index of a country/journal means that they have published H documents and each of which has been cited at least H times. It is usually considered as an indicator to quantify the output and scientific impact of a country or journal.

### Bibliometric and visualized analysis

Three bibliometric tools including CiteSpace (version 5.8.R3), VOSviewer (version 1.6.16) and “bibliometrix” package of R software were used for further bibliometric analysis and drawing scientific knowledge maps. In this study, VOSviewer was adopted to conduct co-citation analysis of journals and co-occurrence analysis of keywords (van Eck and Waltman, 2010; Cheng et al., 2022d; Wu et al., 2022). Co-citation count refers to the frequency with which two papers are cited together by subsequently published papers. While co-occurrence count is defined as the number of works where they occur together and weighted by the frequency of occurrence (Wu et al., 2022). The specific parameters of VOSviewer accept the default settings itself. This software is able to construct different visual maps including the network, density and overlay visualization maps. In general, different maps with various nodes and lines represent different meanings, which have been described in detail in the figure legends.

CiteSpace was mainly used to create co-authorship analysis of countries/institutions, co-cited reference cluster and burst analyses (Synnestvedt et al., 2005; Sabe et al., 2022). Co-authorship patterns are often used in bibliometric studies as an indicator for understanding collaboration. It refers to the evaluation of the relationship among countries/institutions based on the number of coauthored documents (Synnestvedt et al., 2005). CiteSpace adopts several structural metrics of co-authorship/co-citation networks, which include betweenness centrality (BC), modularity, and silhouette. The BC metric generally measures the degree of one node in the middle of a path that connects to other nodes in the network. And it is an indicator to reflect the node’s centrality in the network based on Freeman’s betweenness centrality metric (Freeman, 1977; Chen et al., 2012). These nodes with high centrality are often located in the center of the network, which could regard as the transitions of other parts. In general, nodes with a BC value more than .1 are key hubs that bridges the nodes it links and also displayed with bright red or bright purple rings (Sabe et al., 2022). In co-cited reference cluster, the modularity value measures the extent to which a network can be divided into different modules. And the silhouette is a method of validation of consistency within clusters of data. When the modularity value is greater than .3, the cluster structure is generally considered as significant. While the silhouette value of a network exceeds .3, .5, or .7, the cluster is considered homogenous, reasonable, or highly credible, respectively (Sabe et al., 2022). Burst detection, developed by Kleinberg (Kleinberg, 2003), was applied to detect keywords/references that had a surge of their appearance for a specific period of time. The parameters of CiteSpace were set as follows: 1) time span (2000 to 2021); 2) selecting “1 or 2” as year of slice; 3) selection criteria (top 50); 4) text processing: selecting title, abstract, author keywords, and keywords plus; 5) pruning: selecting Pathfinder and Pruning sliced networks.

Moreover, the “bibliometrix” package of R software was used to conduct country collaboration analysis (Aria and Cuccurullo, 2017). In this map, color shades were correlated with the number of studies published, and the thickness of the line reflected the strength of cooperation.

## Results

### Annual publication and citation trends

**FIGURE 2:**
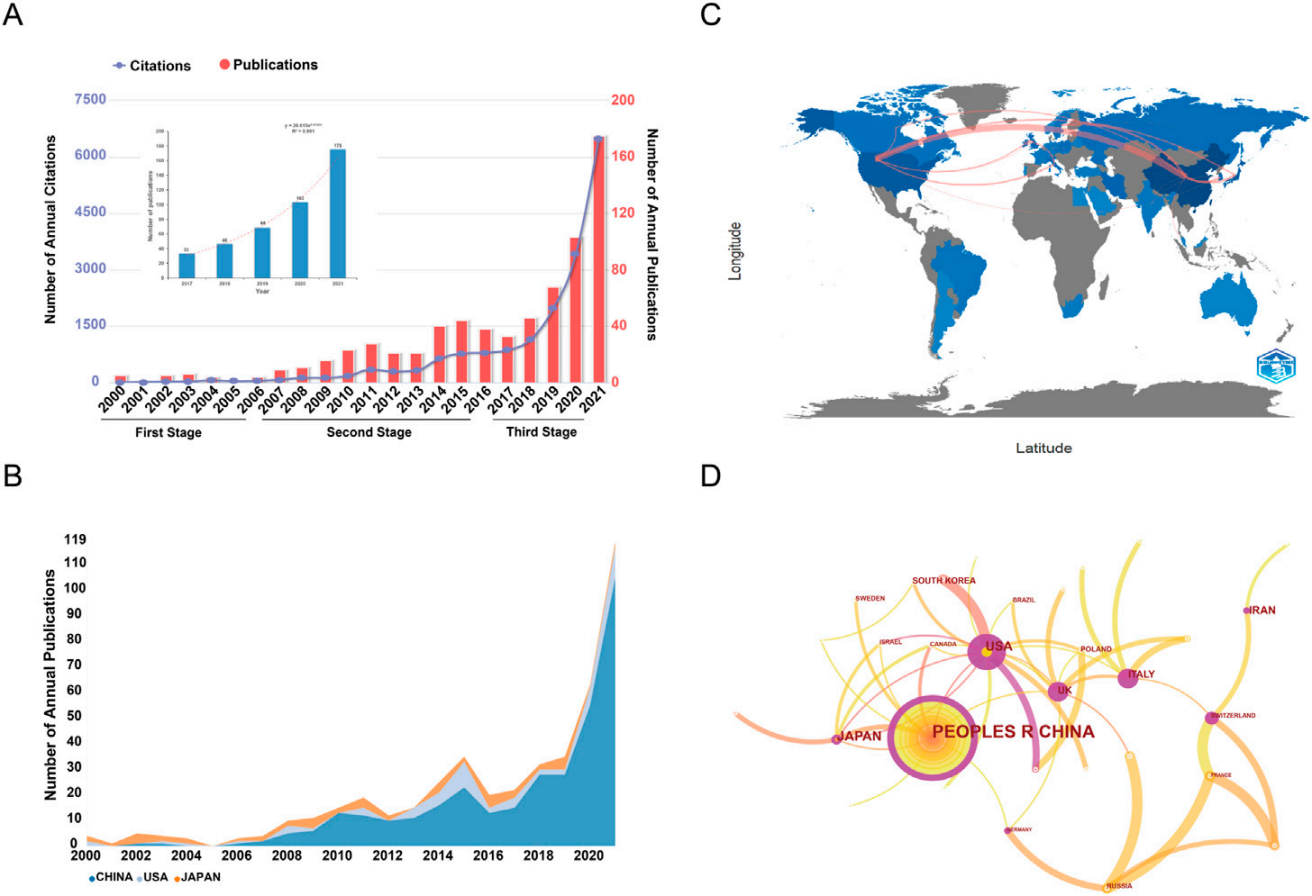
**(A)** Annual publication and citation trends of literature related to SDT. **(B)** Annual publication trend among the top three countries from 2000 to 2021. **(C)** Cooperation analysis of countries/regions involved in SDT research. **(D)** The countries/region’s co-authorship overlay visualization map generated by using VOSviewer. The nodes in the graph represented countries/regions, and lines between the nodes reflected the co-authorship relationships. Nodes were marked with different colors according to their average appearing year (AAY). The size of the nodes was proportional to the number of publications.

According to the search strategies mentioned above, a total of 701 publications including 633 original articles and 68 reviews were obtained from WoSCC for the final analysis. The total times cited score was 24,488 (16,334 without self-citations); on average, items were cited 34.93 times. H-index of all the publications was 71. On the whole, the annual numbers of publications and citations exhibited an upward trend over the past 2 decades. The publication output increased from 5 in 2000 to 175 in 2021. The average growth rate of publications from 2000 to 2021 was 18.4%. As shown in Figure 2A, we could roughly divide the research of SDT into three stages: first stage (2000–2006), second stage (2007–2016), and third stage (2017–2021). Model fitting curve revealed a significant exponential growth trend from 2017 to 2021 (*R* ^*2*^ = .991). Correspondingly, the annual number of citations showed a similar increasing tendency. In addition, the correlation between publications and citations was also analyzed, and the results showed a statistically significant correlation (*r* = .984; *p* < .001).

### Major contributors in the field of SDT

#### Countries/regions

A total of 35 countries/regions had published related studies in this field. The top 10 high-output countries/regions were listed in Table 1. China was the most productive country with 463 documents (66.05%), followed by the United States (87; 12.41%), Japan (77; 10.98%), and Iran (31; 4.42%). Figure 2B demonstrated the annual publication trend among the top three countries from 2000 to 2021. An analysis of collaboration among countries/regions was displayed in Figure 2C. It can be seen that China collaborated most closely with the United States and Japan. Figure 2D showed the country co-authorship overlay visualization map. The color of each node reflected the average appearing year (AAY) of each country. From this figure, we could find that countries marked with red colors were the relatively new entrants in this field.

**TABLE 1:** The top 10 countries in research scope of SDT.

Rank	Country	Quantity	% Of 701	H-index	ACI
1	China	463	66.05	62	35.77
2	United States	87	12.41	35	45.20
3	Japan	77	10.98	31	36.03
4	Iran	31	4.42	14	17.48
5	United Kingdom	28	3.99	19	47.75
6	Italy	20	2.85	11	28.65
7	South Korea	19	2.71	14	56.63
8	Poland	12	1.71	8	19.75
9	Russia	11	1.57	7	19.09
10	Czech Republic	10	1.43	6	14.60

ACI, average citations per item.

#### Institutions

**FIGURE 3:**
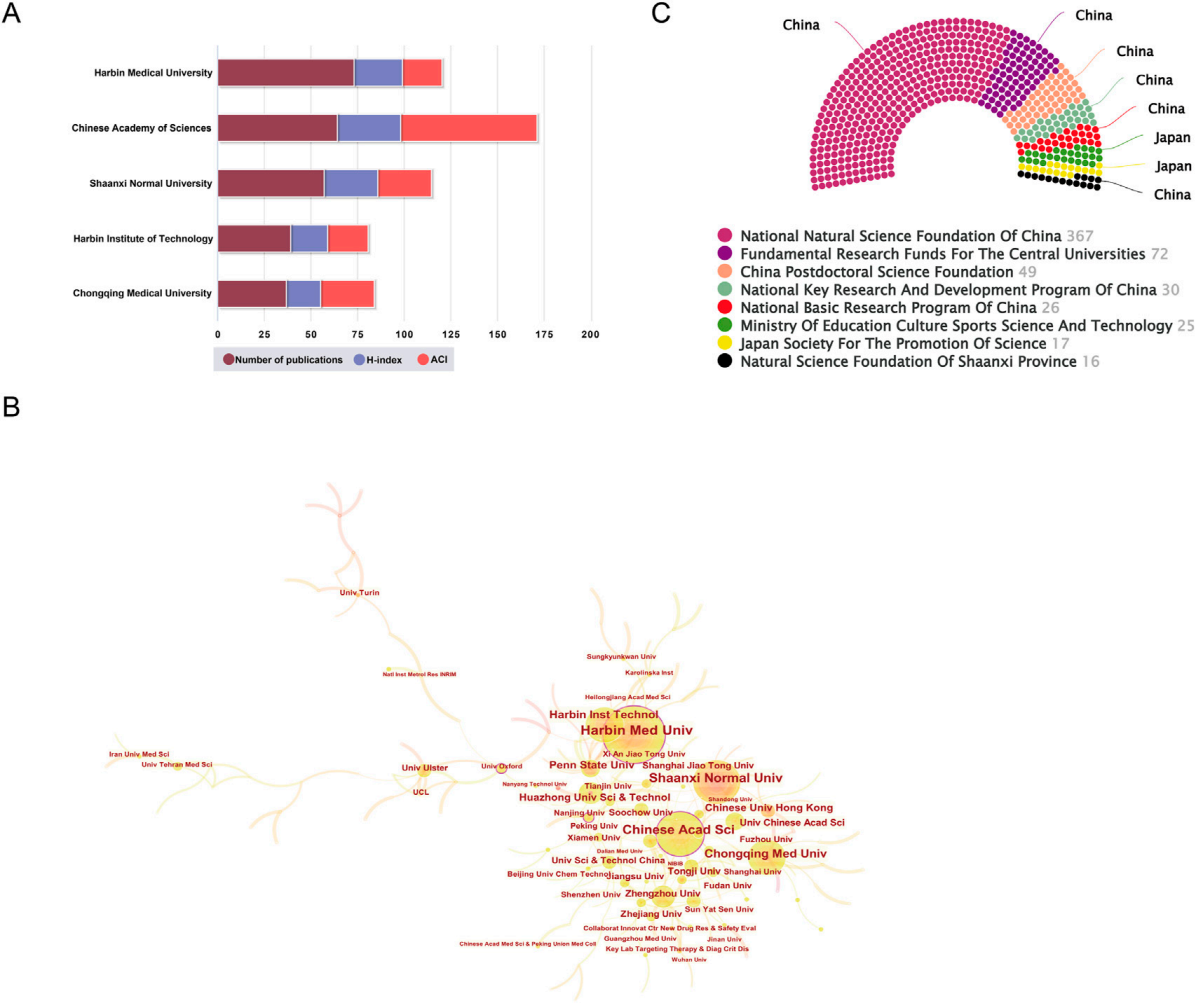
**(A)** The top five most prolific institutions. **(B)** Institutional cooperation analysis by using CiteSpace. In this network map, each node represented an institution, and the node size represented their relative quantity of research outputs. A node with a purple circle showed that the centrality was more than .1. **(C)** The top eight most active funding agencies in this domain.

Figure 3A showed the top five affiliations with the most SDT-related publications. Of them, the highest number of publications was achieved by the Harbin Medical University (N = 73). Chinese Academy of Sciences (N = 64) was the next, followed by Shaanxi Normal University (N = 57). Moreover, Chinese Academy of Sciences had the greatest H-index (N = 34) and the most average citations per item (ACI) (N = 73.06). As for institutional co-authorship analysis in Figure 3B, University of Oxford (.16), Chinese Academy of Sciences (.12), Harbin Medical University (.11), and Nanjing University (.11) occupied the center location of collaboration network, with the BC values in all these institutions greater than .1.

#### Funding agencies

Figure 3C illustrated the top eight related funding agencies for the support of SDT research. From the distribution of funding agencies, the vast majority of these fund organizations were from China. Specifically, the fund project of National Natural Science Foundation of China (NSFC) has sponsored the largest number of studies (of 367 items funded). Fundamental Research Funds for the Central Universities, and China Postdoctoral Science Foundation rank in the second and the third place, with 72 and 49 studies, respectively.

### Most popular journals and research areas

**FIGURE 4:**
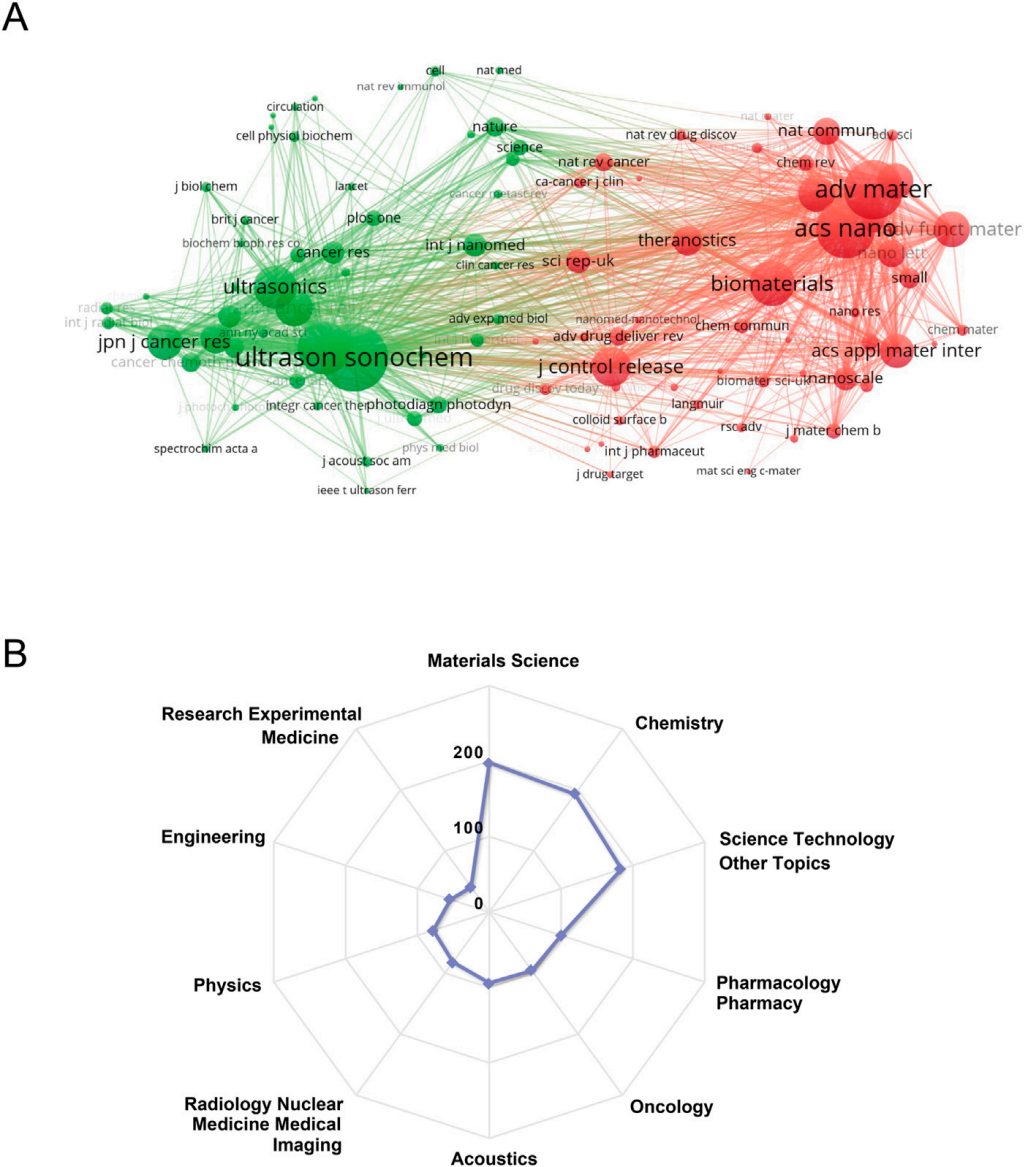
**(A)** Network visualization map of journal co-citation analysis by using VOSviewer. The size of nodes weighted by the amount of citations. **(B)** The top 10 research areas on SDT.

The top 15 most productive journals on SDT were summarized in Table 2. They have contributed to 37.5% of all publications (263 documents). Most of these journals fall into the following WOS categories such as *Acoustics*, *Oncology*, *Chemistry*, *Materials Science*, *Engineering*, and *Nanoscience & Nanotechnology*, etc. Among them, *Ultrasound in Medicine and Biology* published the most papers, followed by *Ultrasonics Sonochemistry*, and *Ultrasonics*. *Ultrasonics Sonochemistry* and *Ultrasonics* had the largest H-index of 19, and *Advanced Materials* was the top-ranked with the highest ACI. According to 2021 JCR report, the top 15 most prolific journals were mainly classified into Q1/Q2 with higher impact factor. As for co-citation analysis, journals with a minimum of 50 citations were included. As shown in Figure 4A, there were 102 nodes and 5114 lines in the network map. The top five most-cited journals were *Ultrasonics Sonochemistry*, *Advanced Materials*, *ACS Nano*, *Ultrasound in Medicine and Biology* and *Biomaterials*. Figure 4B illustrated the top 10 research areas of SDT based on the number of publications. Research areas that received the most interest were *Materials Science*, *Chemistry*, and *Science Technology Other Topics*.

**TABLE 2:** The top 15 most active journals in research scope of SDT.

Rank	Sources title	Quantity	% Of 701	If 2021	H-index	ACI
1	Ultrasound in Medicine and Biology	31	4.42	3.694	18	27.65
2	Ultrasonics Sonochemistry	28	3.99	9.336	19	54.14
3	Ultrasonics	26	3.71	4.062	19	33.92
4	Anticancer Research	23	3.28	2.435	17	36.65
5	ACS Applied Materials Interfaces	19	2.71	10.383	12	29.26
6	ACS Nano	19	2.71	18.027	17	110.32
7	International Journal of Nanomedicine	19	2.71	7.033	10	19.53
8	Biomaterials	17	2.43	15.304	15	45.06
9	Journal of Controlled Release	14	2.00	11.467	13	50.86
10	Advanced Functional Materials	12	1.71	19.924	9	64.75
11	Advanced Healthcare Materials	12	1.71	11.092	9	26.50
12	Photodiagnosis and Photodynamic Therapy	12	1.71	3.577	7	18.33
13	Advanced Materials	11	1.57	32.086	10	118.45
14	Biomaterials Science	10	1.43	7.59	8	27.90
15	Theranostics	10	1.43	11.6	8	40.90

### Keyword Co-occurrence analysis

**FIGURE 5:**
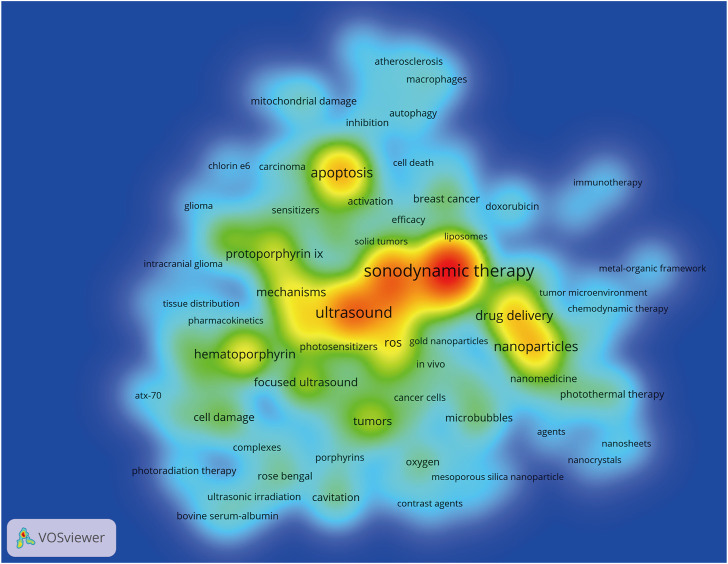
Heatmap of keywords co-occurrence analysis generated by using VOSviewer. The size and color depth of words were positively related to the co-occurrence frequency.

**FIGURE 6:**
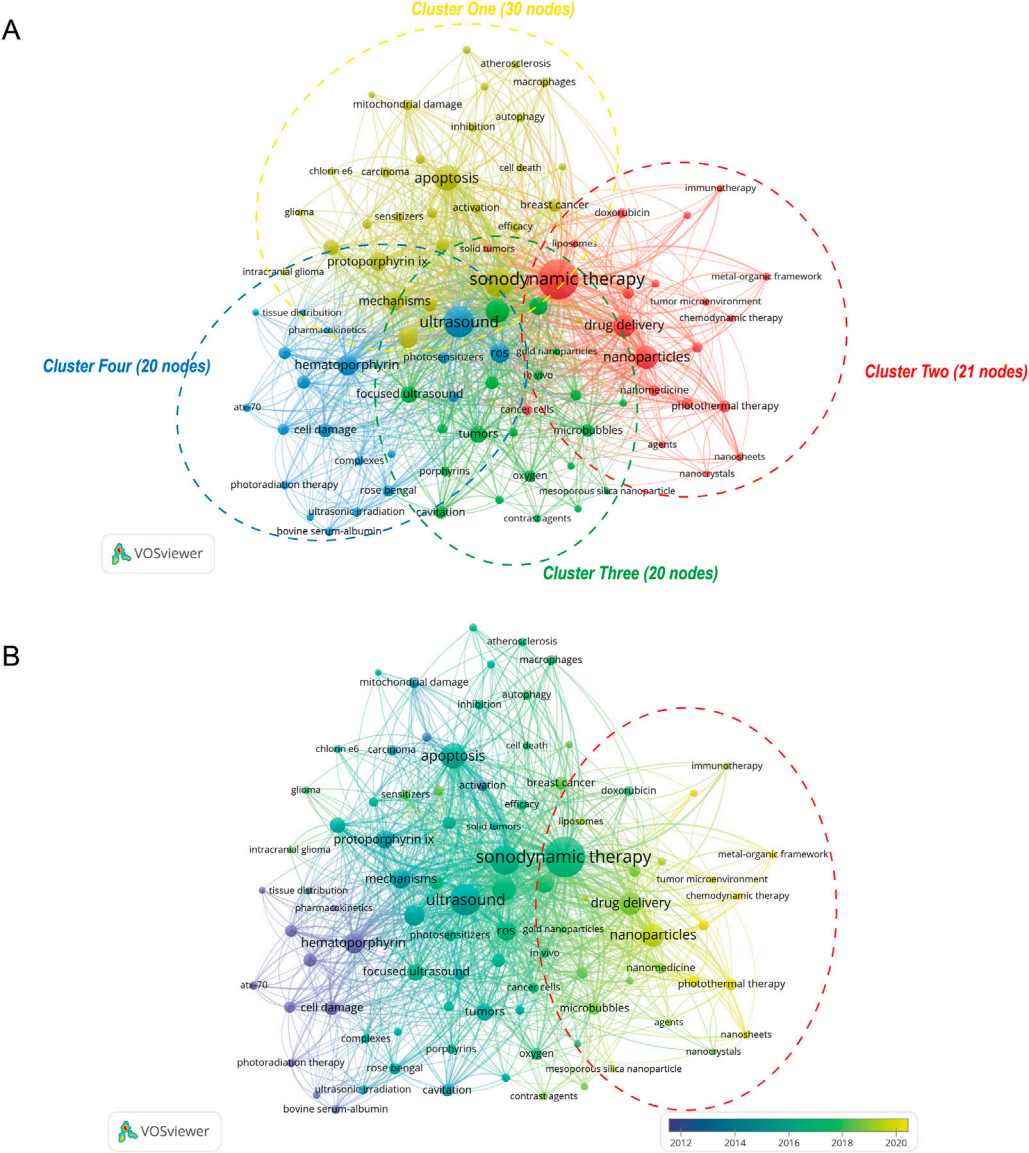
**(A)** Network visualization map of keywords co-occurrence analysis. All the keywords were divided into four different clusters in accordance with different colors. **(B)** Overlay visualization map of keywords co-occurrence analysis. In this panel, the distribution of keywords was presented based on their AAY. The purple and blue colors represented an early appearance, and the red color represented a late appearance.

**FIGURE 7:**
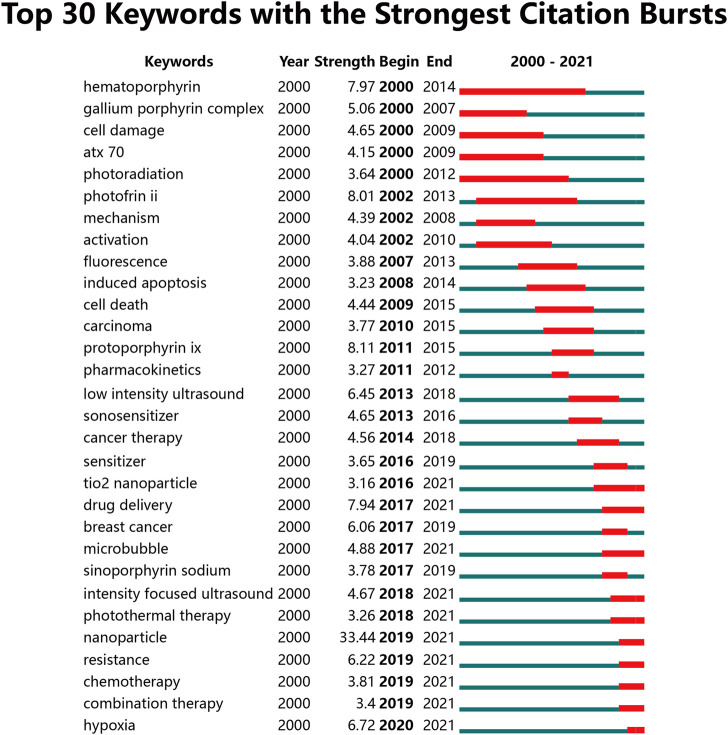
Top 30 keywords with the strongest citations bursts by CiteSpace. The red bars represented the starting and ending, and the duration of citation bursts.

After merging keywords with the same meaning, 91 keywords were obtained from these 701 publications. The heatmap in Figure 5 presented keywords with more than 10 occurrence times. The top 30 most frequent occurrences keywords were summarized in Table 3. Of them, sonodynamic therapy (501 times), ultrasound (281 times), photodynamic therapy (245 times), apoptosis (180 times), and nanoparticles (155 times) were the top 5 keywords with more than 150 occurrences. Additionally, the network visualization map of keywords co-occurrence analysis was shown in Figure 6A. Each color represented the merging of clusters corresponding to the keywords with close connection. As can be seen, all the keywords were divided into four different clusters as follows: cluster 1 (yellow nodes, 30 keywords, studies on mechanisms), cluster 2 (red nodes, 21 keywords, studies on drug delivery and nanoparticles), cluster 3 (green nodes, 20 keywords, studies on cancer therapy), and cluster 4 (blue nodes, 20 keywords, studies on ultrasound and sonosensitizers). Moreover, apart from the network map, we also provided an overlay visualization map in which keywords were colored based on their AAY. As shown in Figure 6B, from a holistic perspective, keywords in cluster t2 have emerged more recently, which implied that these research directions have received much attention from researchers recently. More specifically, keywords with AAY after 2019 include: “immunogenic cell death (AAY = 2020.67)”, “chemodynamic therapy (AAY = 2020.67)”, “nanosheets (AAY = 2020.56)”, “metal-organic framework (AAY = 2020.50)”, “hypoxia (AAY = 2019.94)”, “photothermal therapy (AAY = 2019.81)”, “tumor microenvironment (AAY = 2019.69)”, “mesoporous silica nanoparticles (AAY = 2019.50)”, “nanoparticles (AAY = 2019.39)”, “liposomes (AAY = 2019.27)”, “*Staphylococcus aureus* (AAY = 2019.20)” and so on. Keywords Burst Analysis; Keywords burst was defined as those acquired a great deal of attention for a period of time. The burst duration was set to at least 1 year in CiteSpace, from which we detected the top 30 keywords with the strongest citations bursts (Figure 7). Among them, keyword with the strongest burstiness was nanoparticle (burst strength of 33.44). In addition, the following keywords including “TiO_2_ nanoparticle”, “drug delivery”, “microbubble”, “intensity focused ultrasound”, “photothermal therapy”, “nanoparticle”, “resistance”, “chemotherapy”, “combination therapy”, and “hypoxia” were in burstiness until 2021, which implied that these research directions may continue to explode in the future.

**TABLE 3:** The top 30 most frequent occurrences keywords in research scope of SDT.

Rank	Keywords	Occurrences	AAY	Rank	Keywords	Occurrences	AAY
1	Sonodynamic therapy	501	2016.80	16	5-aminolevulinic acid	63	2015.57
2	Ultrasound	281	2015.12	17	Combination therapy	51	2016.71
3	Photodynamic therapy	245	2016.25	18	Cell damage	50	2011.52
4	Apoptosis	180	2015.62	19	Gallium-porphyrin complex	44	2011.41
5	Nanoparticles	155	2019.39	20	Breast cancer	43	2018.14
6	Cancer	150	2016.94	21	Cancer therapy	42	2016.45
7	Drug delivery	140	2018.51	22	Microbubbles	40	2018.40
8	Hematoporphyrin	115	2012.13	23	Cavitation	38	2014.11
9	*In vitro*	109	2015.30	24	Low-intensity ultrasound	38	2016.11
10	Ros	101	2016.77	25	Photofrin	35	2011.20
11	Mechanisms	97	2014.49	26	Chemotherapy	33	2018.52
12	Protoporphyrin ix	88	2014.45	27	Oxygen	33	2016.97
13	Sonosensitizers	81	2017.44	28	Photothermal therapy	32	2019.81
14	Focused ultrasound	74	2016.35	29	Hypoxia	31	2019.94
15	Tumors	73	2015.48	30	Sarcoma	31	2010.84

AAY, average appearing year.

### Co-cited references and burst analysis

References co-citation analysis was another way to analyze the knowledge base and trace developmental process in a certain research field. In this study, reference co-citation analysis was conducted by CiteSpace and visualized their timelines in Supplementary Figure S1A. All references could be clustered into 15 subclusters with good homogeneity (modularity value = .8228; mean silhouette value = .95). As can be seen that, the cluster of porphyrin-induced sonodynamic effect (#0) was the largest, followed by thp-1 macrophage (#1) and focused ultrasound (#2). Moreover, the evolution characteristics of each cluster could be known from this timeline view. Of note, enhanced sonodynamic cancer therapy (#4) and nanoparticles (#12) have been the hot topics of SDT research in recent years and still continue to be. In addition, the top 30 references with the strongest citations bursts were shown in Supplementary Figure S1B.

## Discussion

### The global research dynamics of SDT

Over the past years, as a safe, effective, low cost and non-invasive approach, SDT has attracted increasing attention in the medical field, particularly for tumor therapy (Yumita et al., 1989). As expected, the annual number of studies on this topic exhibited an upward trend and has increased from 5 in 2000 to 175 in 2021. The developing process of SDT has went through three stages including the initial developing stage (2000–2006), the slow development stage (2007–2016), and the rapid growth stage (2017–2021) in accordance with the growth pattern of literature. Of note, model fitting curve revealed a significant exponential growth trend in the third stage and the year 2017 represented a turning point in the number of published papers. As mentioned earlier, this result can’t be separated from the rapid advances in biomaterial science and ultrasonic medicine (Uthaman et al., 2021a). With growing requirements of cancer treatment, as well as the continuous development of nanotechnology worldwide, one may speculate that more studies on this topic will be published in the foreseeable future. In addition, as we all know that SDT has been proposed as an analogous strategy to PDT and evolved on the basis of the latter. Nevertheless, compared with several bibliometric studies on PDT, there is a substantial gap in total number of publications (Cramer and Chen, 2020; Cheng et al., 2022c; Zhan et al., 2022). This result might mainly be associated with the earlier development of PDT.

### The main contributors from the level of countries, institutions and funding agencies

From the perspective of national contribution, China ranked first in the world in terms of outputs (66.05%). Another bibliometric research also revealed that China had published the most publications with 2145 documents (36.96%) in the field of PDT for cancer treatment (Cheng et al., 2022c). The results above indicate that China has attached great importance to scientific research in these two areas. With regard to research institutions, the contribution of scientific institutions fundamentally represents a country or region. Our result showed that the top five prolific institutions were all located in China. As for the distribution of funding agencies, the vast majority of these fund organizations were from China and the fund project of NSFC has sponsored the largest number of studies. Here, it wasn’t hard to see that China contributed the largest number of studies in this field couldn’t separate from a large number of experienced academic institutions and adequate funding support (Farrokhyar et al., 2016). Additionally, recent epidemiologic evidence suggested cancer incidence and mortality have been growing rapidly year by year worldwide. As the most populated country, the cancer incidence and mortality rate in China are the highest in the world (Bray et al., 2018). To address this challenge, Chinese government has invested tremendous resources in this field, and an increasing amount of Chinese scholars also focused on cancer-related researches (Dou, 2020). Multiple bibliometric studies have confirmed the leading edge in the area of oncology (Klingelhöfer et al., 2019; Cheng et al., 2022e). However, it worth noting that although China has the largest number of publications and the highest H-index, the mean number of citations was lower than other countries such as the United States, Japan, United Kingdom, and South Korea. Although Chinese scholars have published considerable quantity of documents, the quality of these papers literally needs further improvement. Notably, the Chinese Ministry of Science and Technology has noticed this issue and developed a range of relevant measures to encourage researchers to publish more high-quality studies.

### The most popular journals and research areas in the field of SDT

Generally speaking, international peer-reviewed journals are important vehicles to establish effective scientific communication for researchers from different countries (Li et al., 2022b; Xia et al., 2022). Journal statistics could help scholars understand how SDT related information is circulated across academic journals and also provide value references to choose suitable journals for their works. In this study, *Ultrasound in Medicine and Biology*, *Ultrasonics Sonochemistry*, and *Ultrasonics* are the main journals publishing work on SDT. As for journal co-citation analysis, the top five most-cited journals were *Ultrasonics Sonochemistry*, *Advanced Materials*, *ACS Nano*, *Ultrasound in Medicine and Biology* and *Biomaterials*. Of note, these journals share common features, that are high IF. It is generally known that journals with high IF are attracted by scholars in selecting for publishing their high-quality studies, while these papers, in turn bring more citations for journals to further improve their influence (Wu et al., 2021c; Zhang et al., 2022a; Shen et al., 2022b). From the perspective of national distribution of these journals, we have found that there were very few influential journals from East Asia, despite Eastern Asian countries such as China, Japan and South Korea were all the major contributors to this field. This result suggests that there is a need for Asian countries to improve and strengthen the development of international journals to further expand their academic influence of SDT field.

### The main research directions and focuses in current work

Keywords are usually extracted from publications to represent the core content of a given topic, which may include the key messages of research objects, methods, and results (Wu et al., 2021d; Wu et al., 2021e; Yeung et al., 2021). In bibliometrics, keyword co-occurrence analysis is the most powerful way to identify the hot topics of research in a certain field, and keywords-formed clusters could reflect the composition of main research directions and their evolutionary trend. As can be seen from Figure 6B, all the keywords were divided into four different clusters.

Cluster 1 (studies on mechanisms) was the largest cluster including 30 keywords, and the primary keywords were mechanism, *in-vitro*, apoptosis, autophagy, oxidative stress and cell death. A variety of evidences have demonstrated that multiple mechanisms, including ultrasonic cavitation effect, mechanical effect, oxidative stress by generating reactive oxygen species (ROS), enhancement of antitumor immunity, induce tumoral cells died from apoptosis, necrosis, or other forms of cell death (Rosenthal et al., 2004; Bai et al., 2012; Zhu et al., 2022). Take autophagy as an example, it is highly conserved cellular “self-eating” process through which denatured cytoplasmic proteins and damaged organelles are engulfed by autophagosomes. Several previous studies revealed the occurrence of autophagy activation through morphological observation and biochemical analysis after SDT (Zhang et al., 2022b). However, SDT-induced autophagy might act as a double-edged sword. During SDT, the tumor cells experience various environmental stresses, autophagy could provide an alternate energy source and enhance the survival fitness of tumor cells, resulting in reduced therapeutic efficacy. In view of this, many scholars suggested that SDT in combination with autophagy inhibitors could contribute a regimen to the efficiency of tumor therapy (Wang et al., 2010; Feng et al., 2019; Wang et al., 2022a). For example, Wang et al. (Wang et al., 2010) conducted a study to determine role of autophagy in SDT-induced cytotoxicity in murine sarcoma 180 cells and also its relationship with apoptosis by performing inhibitor studies. The results of this study indicated that the combination of SDT with autophagy inhibitors such as 3-methyladenine and Bafilomycin A1 could can significantly enhance the anti-tumor effect of SDT through induction of apoptosis and necrosis. Therefore, further studies elucidating the mechanisms underlying the role of SDT in cell death may contribute to the development of effective strategies in tumor therapy.

Cluster 2 mainly focused on drug delivery and nanoparticles. From a holistic perspective, most keywords in cluster 2 have emerged more recently, which implied that these research directions have received much attention from researchers recently. It is clear that most of the keywords in cluster 2 was associated with nanoparticles or nanomaterials, indicating that nanomaterials-related studies have received great attention in SDT research recently (Uthaman et al., 2015). Nanoparticles are solid colloidal particles with diameters ranging from 10 to 1000 nm. In recent years, a large number of studies have reported nanoparticles carried agents/molecules including chemotherapeutic drugs, nuclides, and siRNA could combine with chemotherapy, radiotherapy, photothermal therapy, or gene therapy to significantly enhance the anti-tumor therapeutic effect (Gong et al., 2019; Chen et al., 2020; Uthaman et al., 2021b; Yang et al., 2022). The application of nanoparticles in SDT mainly includes two directions: nanoparticle carriers and nanoparticle-based sonosensitizers (Huang et al., 2017; Jin et al., 2020; Ren et al., 2022). The former acts as carriers for delivering organic sonosensitizers, which could overcome the inherent shortcomings of small-molecule sonosensitizers. By modulating the spatial structure and chemical composition of nanoparticle carriers, various sonosensitizers or agents with different size, charge, or hydrophobicity could be packaged. In addition, under the cover of these carriers, the adaptability of sonosensitizers to external stimuli is enhanced, which could improve the tissue distribution and enhance drug enrichment in the tumor tissues with a long time (Pan et al., 2018a). As for nanoparticle-based sonosensitizers, nanoparticles themselves may act as sonosensitizers. Among them, the following nanoparticles including Fe_3_O_4_ nanoparticles (Shen et al., 2015), gold nanoparticles (Shanei and Akbari-Zadeh, 2019), as well as porous silicon nanoparticles (Osminkina et al., 2014) have been widely studied. Nevertheless, there is still a long way to go to translate experimental research into clinical practice.

As for Cluster 3 (studies on cancer therapy), the prominent keywords were cancers, tumors, and cancer therapy. Although mounting investigations have reported the roles of SDT non-oncologic disorders such as atherosclerosis and infectious diseases, the application of SDT currently attracting the most attention is still tumor therapy, especially for solid tumors (Pang et al., 2020; Chen et al., 2021; Li et al., 2021). A large number of studies have demonstrated the positive results regarding the use of SDT in different tumor animal models. A previous study by our group also demonstrated the efficacy of SDT for glioblastoma treatment (Wang et al., 2022b). However, relatively speaking, the research on SDT is lagging and most studies on this topic are still in the pre-clinical stages of research. As of date, PDT has already been used in the clinic for cancer treatment and an increasing number of multicentric studies and clinical trials are in progress (Cramer and Chen, 2020). Therefore, to further facilitate the translation of this technology to clinical use, more in-depth research is necessary.

Cluster 4 includes 20 keywords, mainly related to studies on ultrasound and sonosensitizers. As outlined previously, SDT consists of three essential components that is ultrasound, sonosensitizer and oxygen molecules (Trendowski, 2014; Rengeng et al., 2017). Of them, the choice of an appropriate sonosensitizer with tumor-targeting capability becomes important. Currently, most of the studied traditional sonosensitizers such as porphyrins, xanthene and 5-aminolevulinic acid are from photosensitizers (Chen et al., 2014; Pang et al., 2016). Recently, our research group has innovatively discovered that temozolomide, as the first‐line chemotherapeutic agent to treat malignant glioblastoma, exhibited significant sonosensitivity, which could provide a potential breakthrough and opens a door for clinical treatment of glioblastoma (Wang et al., 2022b). Nevertheless, most of these sonosensitizers has short half-life, poor *in vivo* targeting and water solubility, making it difficult to be enriched at the tumor site. With remarkable advancements in nanotechnology, nanomaterials have been introduced as an alternative route to overcome the above issues of traditional sonosensitizers and increasing number of nano-sonosensitizers have been designed and widely used recently (Guo et al., 2022). As for ultrasound, studies mainly concentrated on ultrasound setting parameters including frequency, intensity, transducer geometry, coupling media, as well as other experimental conditions that could affect reproducibility and transferability (Secomski et al., 2017; Araújo Martins et al., 2021; Tamboia et al., 2022). However, to our knowledge, the ultrasound parameters in different laboratories are still not fully standardized. In addition, due to large differences between *in vivo* and *in vitro* experiments, translation of ultrasound conditions from *in vitro* to *in vivo* is still a bottleneck in the SDT field.

### The potential research hotspots and frontiers in the future

Apart from keywords cluster analysis, VOSviewer provided an individualized analysis for each keyword according to their average appearing years in all documents. The purple and blue colors represented an early appearance, and the yellow color represented a late appearance. Similar to VOSviewer, CiteSpace has a unique function of burst detection. This is generally acknowledged as an effective tool to capture the sharp increased attention on keywords within a certain period. And these keywords with an ongoing burst until now deserve special attention because they may have great potential to continue to be hot topics in the near future (Wu et al., 2021a). Taken together with keywords co-occurrence and burst analysis, with the exception of nanomaterials-related studies including nanoparticles, mesoporous silica nanoparticles, nanosheets, liposomes, microbubble and TiO_2_ nanoparticle, the following research directions such as immunogenic cell death (Zheng et al., 2021; Zheng et al., 2022), metal-organic framework (Pan et al., 2018b), photothermal therapy (Liang et al., 2019), hypoxia (Chen et al., 2017; Zhu et al., 2018), tumor microenvironment (An et al., 2020), chemodynamic therapy (Bai et al., 2020), combination therapy (Ning et al., 2022), tumor resistance (Guan et al., 2022), intensity focused ultrasound (Ohmura et al., 2011; Roberts et al., 2022), drug delivery (Ren et al., 2022), and *Staphylococcus aureus* (Yu et al., 2021) also deserve further attention and may continue to explode in the future. Given space limitations, we only discussed several directions such as immunogenic cell death (ICD), hypoxia and *Staphylococcus aureus* as follows.

### ICD

ICD is a particular modality of cell death characterized by the upregulation of various damage-associated molecular patterns (DAMPs), and generally be triggered by several chemotherapeutic agents such as oxaliplatin, anthracyclines, and paclitaxel (Kroemer et al., 2022). Increasing studies have found that SDT is capable of inducing ICD in multiple cancer types and promises to play an assistive role in tumor immunotherapy (Zheng et al., 2021; Zheng et al., 2022). For example, Wan and colleagues have designed a nuclear-targeting delivery system TIR@siRNA to achieve the gene augmented nuclear-targeting SDT. The results demonstrated that this enhanced SDT strategy could induce ICD to improve the immunosuppressive microenvironment, thus boosting anti-programmed cell death-ligand 1 (PDL-1) therapy for colorectal cancer (Wan et al., 2021). Therefore, this produced ICD effect of combining SDT may offer a new dimension to facilitate immune checkpoint blockade therapy in the future.

### Hypoxia

ROS are a family of highly reactive molecules derived from the metabolism of oxygen. SDT is capable of utilizing ultrasound activated sonosentizer to transfer its excited-state energy to the surrounding oxygen, eventually leading to excessive production of ROS and cellular oxidative damage (Chen et al., 2017; Zhu et al., 2018). However, hypoxic microenvironment is an important feature of solid tumor, which hinders the sonodynamic response *in vivo*. Additionally, SDT-mediated oxygen consumption further exacerbates hypoxic condition, potentially causing a variety of adverse consequences, such as tumor progression, immunosuppression, inflammation, invasion, and metastasis (Lu et al., 2022). In order to overcome these limitations caused by hypoxia, multiple strategies that mainly categorized into “oxygen-generating” or “oxygen-carrying” methodologies have been proposed. For example, reactive oxygen-generating materials including hydrogen peroxide enzymes, metal oxides, and active nanoparticles are able to react with hydrogen peroxide or acid *in vivo* to produce oxygen (Tao et al., 2022). Moreover, through the use of oxygen-carrying microbubbles or nanocarriers such as perfluorocarbons and hydrocarbons, they could release amounts of oxygen to improve oxygen supplementation in tumor site (Lu et al., 2022). Therefore, the development of strategies to relieve tumor hypoxia for improved SDT efficacy will be a very important research area in the future.

### Staphylococcus aureus

Aside from the field of tumor treatment, SDT also has applications in many other fields. Several studies have found that SDT had significant bactericidal activity against bacterial infections, and even methicillin-resistant *Staphylococcus aureus* (Yu et al., 2021). Meanwhile, as PDT is limited by the inability of light to penetrate through human tissue, SDT that could overcome this limitation, exhibit great potential to address the problem of deep tissue bacterial infection such as osteomyelitis, otitis media, and periprosthetic infection (Su et al., 2020; Feng et al., 2022; Su et al., 2022).

### Strengths and limitations

To our knowledge, this is the first bibliometric analysis dedicated to assess the global research trend on SDT. In this study, three bibliometric tools including CiteSpace, VOSviewer, and R-bibliometrix were combained to perform this analysis, which could offer a more comprehensive knowledge landscape for this technology. However, this study does have a number of inherent limitations. Firstly, the literature search only started from 2000. Nevertheless, as our findings illustrated that most of documents related to SDT were published in recent years, we believe that this limitation will have minimal impact on our findings (Yeung et al., 2022; Zhou et al., 2022). Secondly, because of a limitation of these bibliometric software, it is hard to merge data from different databases. Thus, we only analyzed records from WoSCC database, which could inevitably miss some related studies in other databases. Nevertheless, previous studies indicated that is WoSCC was one of the most trustworthy and most used databases for bibliometric analysis (Li et al., 2022c; Cheng et al., 2022f; Song et al., 2022). In addition, given the fact that the citation times of one study usually reach a peak in 3–10 years after publication, another limitation is that the current analysis wasn’t able to underscore the recently published studies.

## Conclusion

This study has summarized the current status and global trends on SDT through bibliometric approach. China was the leading country in contribution and influence in this field. The number of publications on SDT is still in the stage of rapid growth. Furthermore, nanomaterials-related studies have received great attention in SDT research recently. Several other research directions such as immunogenic cell death, metal-organic framework, photothermal therapy, hypoxia, tumor microenvironment, chemodynamic therapy, combination therapy, tumor resistance, intensity focused ultrasound, drug delivery, as well as *Staphylococcus aureus* also may be potential hotspots for future research and deserve further attention. All in all, this bibliometric analysis could provide valuable references for researchers, especially new entrants, as it could help them comprehensively understand the knowledge landscape including main contributors and active journals in the field, as well as find inspiration from the analysis of research hotspots and frontiers in the future.

## Data Availability

The original contributions presented in the study are included in the article/Supplementary Material, further inquiries can be directed to the corresponding authors.
