# Supplementary material for: Mapping knowledge landscapes and emerging trends of sonodynamic therapy: A bibliometric and visualized study
Source: Front Pharmacol. 2023 Jan 9;13:1048211. doi: 10.3389/fphar.2022.1048211 (PMC9868186; doi:10.3389/fphar.2022.1048211)
Supplement: Supplementary file 1 [file DataSheet1.DOCX]

Supplementary Material

# Supplementary Figures and Tables

## Supplementary Figure


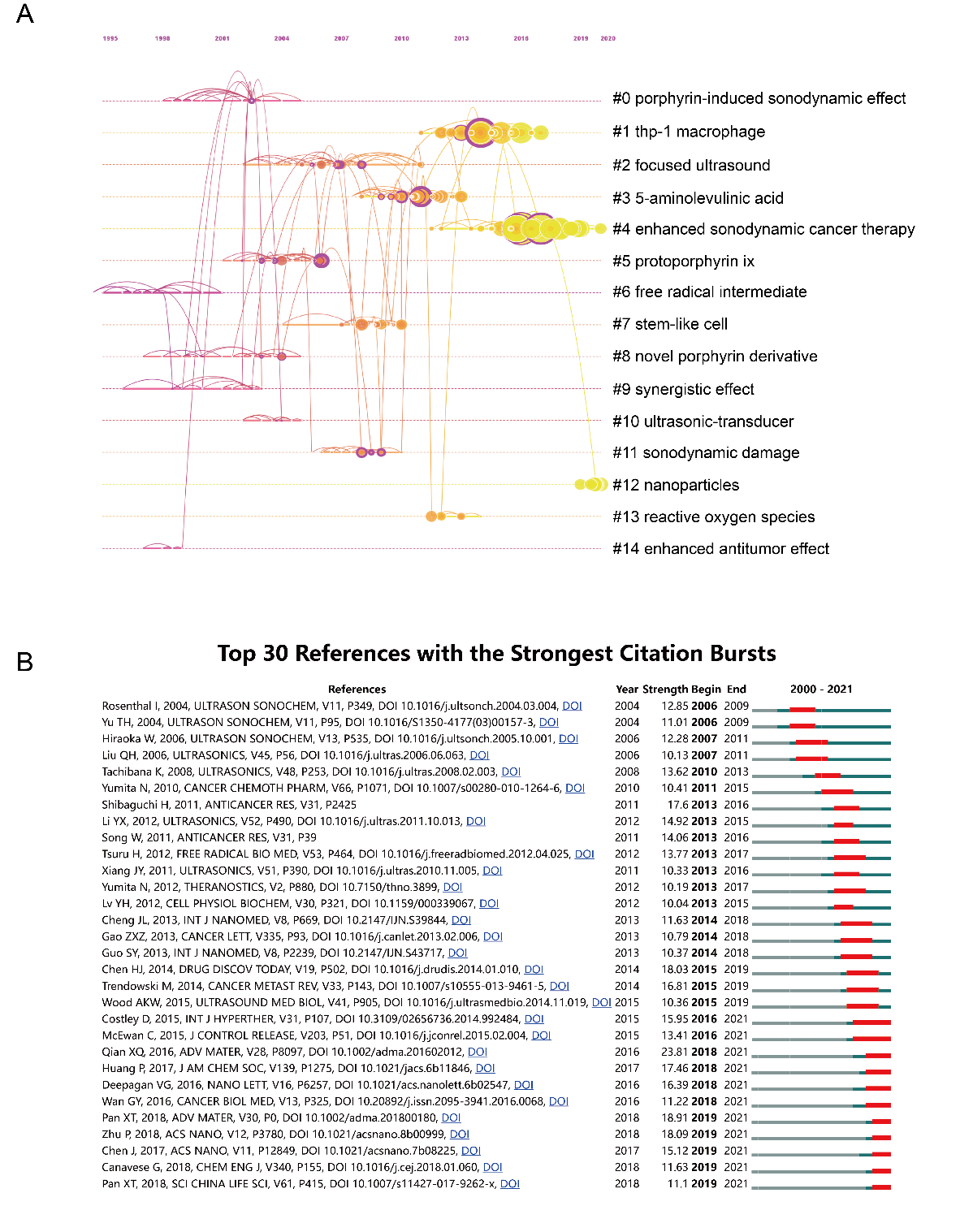


**Supplementary Figure 1.** (A) The timeline view map of co-cited reference clusters. All the references could be clustered into 15 subclusters. Time evolution was indicated with different colored lines. The purple outer circle highlighted nodes with centrality greater than 0.1. (B) Top 30 references with the strongest citations bursts by CiteSpace.
